# Association between Body Mass Index and Migraine: A Survey of Adult Population in China

**DOI:** 10.1155/2018/6585734

**Published:** 2018-04-19

**Authors:** Qingqing Huang, Xiping Liang, Shiqiang Wang, Xiaosong Mu

**Affiliations:** Chongqing University Cancer Hospital & Chongqing Cancer Institute & Chongqing Cancer Hospital, Chongqing, China

## Abstract

Both migraine and obesity are prevalent disorders in the general population, which are characterized by disability and impaired quality of life. Although so many researches had studied the association between migraine and obesity, there are still no full knowledge of the relationship between body mass index (BMI) and migraine, especially chronic migraine (CM). In this study, we analyzed a previous epidemiological survey data of primary headache patients in Chongqing, which surveyed consecutive neurological outpatients through face-to-face interview with physicians using a headache questionnaire. 166 episodic migraine (EM) patients and 134 chronic migraine (CM) patients were included in the study out of 1327 primary headache patients. And 200 healthy adults from the physical examination center were included as a control group. Finally, we found that the patients with migraine (EM and CM) were more likely to be overweight, obese, or morbidly obese compared to those in the healthy group. Significant difference was found between BMI and frequency of migraine attacks but not severity or duration of headache onset. And no significant difference was found in severity and duration of headache onset between episodic and chronic migraine among different BMI classifications. Such may update our knowledge about the clinical features of migraine and BMI, revealing that the frequency of attacks may be associated with being overweight, obese, or morbidly obese in patients with migraine and that the extent of being overweight, obese, or morbidly obese in CM patients was lower than that in EM patients.

## 1. Introduction

Migraine is one of the most frequent consultation syndromes at headache clinics with various neurological, gastrointestinal, and autonomic changes and has a worldwide prevalence which ranges from 5 to 35% in females and from 3 to 20% in males [1]. Based on the International Classification of Headache Disorders (ICHD-II) guidelines, migraine is classified as EM and CM, with CM defined as having 15 or more headache days per month, in which at least 8 days meet the criteria for migraine and lasting for at least three months [2].

Obesity is comorbid with a number of chronic pain syndromes. The relationship between obesity and migraine has been the focus of clinical research in recent years. Previous studies had revealed that obesity was related with migraine patients with high frequency, greater severity, and some increased associated symptoms: higher disability grades, aura and an increased frequency of photophobia and phonophobia [3–5]. Individuals with episodic headache and obesity developed chronic daily headache (CDH) 5 times more than normal-weight individuals [6]. A cross-sectional population study also confirmed the relationship between obesity and CDH and suggested that obesity was a risk factor for transforming from EM to CM [5]. What is more is that the risk of transforming from EM to CM was increased in patients with obesity [7]. Taken together, obesity is not only associated with severity and frequency of migraine attacks but also associated with the chronicity of migraine. But there is little knowledge of the association between other BMI classifications and migraine, especially CM, and why obesity was more apt to CM.

Therefore, we played a second analysis of a previous data, which predicted that different BMI classifications might be associated with EM and CM. We focused on the following questions: (1) the relationship between different BMI and migraine (EM and CM); (2) separately exploring the relationship of BMI and the frequency, severity, and duration of headache onset and their influence on migraineurs; and (3) the link between BMI and chronicity of migraine.

## 2. Methods

This is a second analysis of previous large clinic-based study survey data, which was completed in 2011, conducted at the Neurological Outpatient Department of the First Affiliated Hospital of Chongqing Medical University, China. The target population for this analysis are adults aged 18–65. Younger and older people, as well as pregnant women, were excluded because of their changing weight. The study protocol was reviewed and approved by the Ethics Committee of the First Affiliated Hospital of Chongqing Medical University. Patients were informed of the purpose of the study and provided consent prior to participating.

All patients with a chief complaint of headache were included if they fulfilled “EM” and “CM” criteria according to the ICHD-II criteria. “Frequent” headache was defined as occurring on 10–14 days/month. Each participant was given a questionnaire to record the demographic characteristics, clinical features of the headache (frequency, duration, and intensity), and other information. Electroencephalography (EEG), radiography, transcranial Doppler ultrasound (TCD), and computed tomography (CT) of the brain were carried out if necessary in order to exclude second headache or other diseases.

Pain intensity was assessed on the 11-point pain scale (no pain: 0, mild: 1–3, moderate: 4–6, severe: 7–10). Both body height and weight were measured on subjects with light clothing and without shoes. BMI was calculated as the following formula: BMI = weight (kg)/height (m)^2^. Using WHO guidelines, we defined five categories based on BMI: underweight (<18.5), normal weight (18.5 to 24.9), overweight (25 to 29.9), obese (30 to 34.9), and morbidly obese (35). We combined the data of obese and morbidly obese patients as one group due to the small number of morbidly obese.

## 3. Statistical Analysis

Analysis was performed using SPSS version 17.0 program. Data were summarized using frequency counts and descriptive statistics. Measurement data variables were expressed as means ± SEM. One-way ANOVA tests and *t*-tests were used for comparison when appropriate. A value of *P* < 0.05 was considered statistically significant.

## 4. Results

### 4.1. Demographic Characteristics of the Sample

During the study period (July 20, 2011 to December 30, 2011), 10,315 consecutive patients visited the neurological outpatient of the First Affiliated Hospital of Chongqing Medical University. 1327 patients (12.9%) cited headache as their chief complaint, 166 of these headache patients were diagnosed as having EM (128 women and 38 men; mean age ± SD: 40.42 ± 2.38 years), and 134 were diagnosed as having CM (104 women and 30 men; mean age ± SD: 43.36 ± 2.93 years). At enrollment, the distribution of demographic variables was shown in Table 1.

### 4.2. Proportion of BMI Classifications in Migraine

The proportion of BMI classifications in three groups were shown in Table 2. The normal-weight subjects were more common than the other classifications in the three groups (67.5% for control versus 72.3% for EM versus 69.4% for CM), but no significant difference was found between them. And the underweight subjects showed no difference in the three groups (*P* > 0.05). Compared to the healthy group, both EM and CM patients had a similar trend; they were less likely to be overweight, obese, and morbidly obese; while when compared with that in EM patients, the proportion of overweight, obese, and morbidly obese in CM patients was significantly higher (*P* < 0.05). In other words, CM patients were more likely to be overweight, obese, or morbidly obese than EM patients.

### 4.3. Pain Intensity and BMI

We further divided all migraineurs into three groups according to pain severity: mild (1–3), moderate (4–6), and severe (7–10). The results showed that the proportion of four different BMI were not significantly different in three pain severity groups neither in EM nor in CM patients (*P* > 0.05) (Tables 3 and 4). So, maybe we can conclude that pain severity had no correlation with BMI and will not influence the BMI level.

### 4.4. Duration of Headache Onset and BMI

Most migraine patients had gastrointestinal symptoms, such as nausea and vomiting, which would influence their appetite. As a result, we analyzed whether the duration of headache onset had an effect on the BMI level. Migraineurs were well distributed into three groups (<4 h, 4–72 h, and >72 h) according to the duration of headache onset. The data showed that the proportion of four BMI classifications had no difference between different durations of headache onset in the groups (*P* > 0.05). Both in EM and in CM, the duration of headache onset had no effect on the BMI level. The results were shown in Table 5 for EM and Table 6 for CM.

### 4.5. Frequency and BMI

Our study had revealed that CM patients were more apt to be overweight, obese, or morbidly obese than EM. We further compared the relationship of BMI level and frequency of migraine. We first compared the frequency of migraine attacks in episodic migraine. The result was shown in Table 7. There were 12.7% (21/166) EM patients who had 10–14 headache days per month which was defined as frequent migraine. In the EM group, frequent migraine patients had a less proportion in four BMI classifications than low-frequency migraines (<10 days/month). But we also found that with the increase of BMI level, the proportion of frequency migraine began to increase; meanwhile, the low-frequency migraine presented a negative trend. Maybe, it revealed that the frequency of headache had a positive trend with the increase of BMI. In other words, the more the frequency, the heavier the weight.

### 4.6. Extent of Overweight, Obese, or Morbidly Obese in EM and CM

Finally, we compared four BMI values in the severity and the duration of headache onset and frequency between EM and CM. In the severity and the duration of headache onset, four BMI classifications had showed no significant difference between EM and CM. Whereas, when we compared CM (>15 days/month) with frequent migraine (10–14 days/month, including EM according to ICHD-II), the results showed that CM had a more BMI score in classification of normal weight but less score in overweight and obese/morbidly obese, but only normal weight and overweight had a significance (Table 8, *P* < 0.05).

Considering the result of Table 2, the proportion of overweight, obese, or morbidly obese was significantly lower in migraineurs compared with healthy controls. In migraineurs, CM patients were more apt to be overweight, obese, or morbidly obese than EM patients, but the extent of being overweight, obese, or morbidly obese was lower than EM patients. Further analysis shows that the frequency of attacks of migraine is associated to the higher proportion of being overweight, obese, or morbidly obese in frequent migraineurs.

## 5. Discussion

The relationship between obese and migraine in general has been studied in clinical research in recent years [8]. The main findings of our study are the following: compared with the healthy control group, both EM and CM patients were less likely to be overweight, obese, or morbidly obese; while compared to the EM group, the proportion of being overweight, obese, or morbidly obese was higher in the CM group.

The relationship between obesity and migraine was still controversial. Previous studies had reported that obesity was comorbid with migraine in the severity and frequency of migraine attacks [4, 8, 9]. Obesity has also been found to be associated with CM but not chronic tension-type headache [5, 10]. The study of Bond et al. further revealed that the decrease in BMI is associated with the reduction of migraine and migraine outcomes [11]. The association between BMI and migraine was still confined. Some studies found no significant relationship between migraine and BMI [12–14]. A Swedish cross-sectional study showed that there was no relation between obesity and the distribution of frequency, intensity, duration, or severity of attacks compared to nonobese women with migraine [3].

In our case series, we found that overweight and obese/morbidly obese patients had a higher likelihood of having CM than EM. Consistent with a previous study, we found no significant connection between BMI and the severity and the duration of headache onset but a connection with the frequency of migraine attacks was found [3, 14]. It is worth noting that frequent migraine attacks turn to be easily overweight than low-frequency migraine attacks (<10 days/months) in EM patients.

To further study the relationship of migraine and BMI, we tested four different weight classifications in two migraine groups. The significance between migraine and BMI was related neither with severity nor with the duration of headache onset, but was related with the frequency of migraine attacks. CM patients were more likely to have a higher BMI score in the classification of normal weight, but less BMI score in overweight classification comparing with EM patients. It revealed that the frequency of migraine attacks may be associated to the higher proportion of overweight, obesity, or morbidly obese in chronic migraineurs, but the increase extent of BMI was lower than EM.

A similar result was shown in the study of Mamontov et al. [10]. These refined our knowledge on the clinical feature of episodic or chronic migraine. Maybe, further investigation is necessary to better understand the reason and the mechanisms.

Several mechanisms supported that there may exist a possible link between BMI and headaches. Concentrations of calcitonin gene-related peptide (CGRP) levels, which are elevated in obese individuals [15], are a very important mediator of migraine/chronic migraine, and CGRP inhibitors are effective in the treatment of migraine. Additionally, inflammation has been proposed as a possible contributing mechanism for migraine attacks and obesity itself is proinflammatory by the increase in the concentrations of circulating cytokines [16–18]. Furthermore, migraines, like obesity, have been reported as a risk factor for cardiovascular disorders, as well as for stroke [6, 7, 19]. Finally, migraine prevention medications, which were used in the preventive treatment of migraine, may also be a possible contributing reason for the change of BMI [20, 21].

Our study evaluated the relationship between BMI and CM and the difference between epidemic and chronic migraine among different weight classifications in a clinic-based study. Diagnosis was made by physician interviewers using a questionnaire based on the ICHD-II criteria. All measurements were recorded by interviewers to confirm the right information of body height and weight. However, there are also some limitations that should be addressed here. It was a hospital-based study, and the results may be inappropriate to the general population. Besides, the study sample was limited. Therefore, further studies should be conducted in bigger population in the future.

In conclusion, overweight, obesity, or morbidly obese was associated with the frequency of migraine attacks but not the severity and duration of headache onset. The frequency of headache is connected with BMI changing. The more frequency, the heavier the weight when the headache was not chronic. Once the patient is transforming from EM to CM, the increasing trend of BMI was decreased. But more researches are needed in this field.

## Figures and Tables

**Table 1:** Demographic characteristics of the samples.

Characteristics	Control		EM		CM		Total sample	
	*n*	(%)	*n*	(%)	*n*	(%)	*n*	(%)
Gender								
Male	47	23.5	38	22.89	30	22.38	115	23
Female	153	76.5	128	77.11	104	77.61	385	77
Mean age (year) (mean ± SD)	42.81 ± 0.55		40.42 ± 2.38		43.36 ± 2.93		
Range (year)	18–65		18–65		18–65			18–65
Educational level								
Illiterate	17	8.5	14	8.43	10	7.46	41	8.2
Primary school	38	19	32	19.28	26	15.66	96	19.2
Secondary school	45	22.5	43	25.9	33	24.62	121	24.2
High school	53	26.5	47	28.31	37	27.61	137	27.4
College and above	47	23.5	30	18.07	28	20.9	105	21
Marital status								
Unmarried	35	17.5	29	17.46	14	10.45	78/500	15.6
Married	138	69	109	65.67	93	69.4	340	68
Divorced	17	7	18	10.84	19	14.18	54	10.8
Widowed	10	5	10	6.02	8	5.97	28	5.6

**Table 2:** Proportion and BMI categories.

Body mass index (BMI)	Control		EM		CM		*P*
	*n*	(%)	*n*	(%)	*n*	(%)	
Underweight (<18.5)	15	7.5	16	9.6	13	9.7	0.9343
Normal weight (18.5–24.9)	135	67.5	120	72.3	93	69.4	0.094
Overweight (25–29.9)	39	19.5	25	15.1	23	17.2	0.0193
Obese/morbidly obese (>30)	11	5.5	5	3	5	3.7	0.0008

**Table 3:** No correlation between the severity of headache and BMI was found in EM.

Weight category	Mild		Moderate		Severe		*P*
	*n*	(%)	*n*	(%)	*n*	(%)	
Underweight (*n* = 16)	0	0	9	56.2	7	43.8	0.254
Normal (*n* = 120)	2	1.7	55	45.8	63	52.5	0.259
Overweight (*n* = 25)	1	4	9	36	15	60	0.261
Obese/morbidly obese (*n* = 5)	0	0	4	66.7	1	33.3	0.653

**Table 4:** No correlation between the severity of headache and BMI was found in CM.

Weight category	Mild		Moderate		Severe		*P*
	*n*	(%)	*n*	(%)	*n*	(%)	
Underweight (*n* = 13)	0	0	5	38.5	8	61.5	0.487
Normal (*n* = 93)	3	3.61	43	44.6	50	53.8	0.251
Overweight (*n* = 23)	0	0	12	52.2	11	47.8	0.651
Obese/morbidly obese (*n* = 5)	0	0	2	40	3	60	0.534

**Table 5:** There was no association between the duration of headache attacks and BMI in EM.

Weight category	<4 h		4–72 h		>72 h		*P*
	*n*	(%)	*n*	(%)	*n*	(%)	
Underweight (*n* = 16)	6	37.5	7	43.8	3	18.8	0.2415
Normal (*n* = 120)	25	20.8	84	70	11	9.2	0.8176
Overweight (*n* = 25)	6	24	14	56	5	20	0.0529
Obese/morbidly obese (*n* = 5)	0	0	3	60	2	40	0.2076

**Table 6:** There was no association between the duration of headache attacks and BMI in CM.

Weight category	<4 h		4–72 h		>72 h		*P*
	*n*	(%)	*n*	(%)	*n*	(%)	
Underweight (*n* = 13)	4		5		4		0.2415
Normal (*n* = 93)	21		60		12		0.1025
Overweight (*n* = 23)	8		12		3		0.0529
Obese/morbidly obese (*n* = 5)	1		2		2		0.2076

**Table 7:** Frequency headache was significant associated with BMI.

Weight category	<10 days/months		10–14 days/months		*P*
	*n*	(%)	*n*	(%)	
Underweight (*n* = 16)	16	100	0	0	0
Normal (*n* = 120)	108	90	12	10	<0.0001
Overweight (*n* = 25)	18	72	7	28	0.0006
Obese/morbidly obese (*n* = 5)	3	60	2	40	0.2392

**Table 8:** Frequency of headache was significant among different BMI categories between CM and EM.

Weight classification	Severe			Time of headache onset (>72 h)			Frequency (>10 days/months)		
	EM	CM	*P*	EM	CM	*P*	FM	CM	*P*
Underweight	16.71 ± 0.5884	17.14 ± 0.3642	0.3807	17.82 ± 0.1337	17.77 ± 0.1249	0.4315	0	17.16 ± 0.3904	0
Normal	21.34 ± 0.2356	21.58 ± 0.2831	0.5275	21.44 ± 0.5397	22.22 ± 0.4548	0.2296	21.40 ± 0.3313	21.83 ± 0.2063	<0.0001
Overweight	27.52 ± 0.3692	27.16 ± 0.3680	0.5031	26.17 ± 0.6147	26.34 ± 0.5946	0.1057	27.43 ± 0.3776	27.28 ± 0.2644	0.0001
Obese/morbidly obese	34.22 ± 2.919	34.06 ± 2.729	0.9853	31.35 ± 0.2431	30.85 ± 0.7500	0.3991	38.07 ± 4.157	34.38 ± 0.8696	0.1696

## Abbreviations

BMI: Body mass index
CM: Chronic migraine
EM: Episodic migraine
ICHD-II: The International Classification of Headache Disorders
FM: Frequency migraine.
